# Somatosensory and visual evoked potentials and brainstem auditory evoked responses in osteoarthritic cats with chronic pain – a comparative study

**DOI:** 10.3389/fvets.2026.1794107

**Published:** 2026-04-10

**Authors:** Aliénor Delsart, Aude Castel, Maria Vanore, Colombe Otis, Maxim Moreau, Frédéric Sauvé, Bertrand Lussier, Johanne Martel-Pelletier, Jean-Pierre Pelletier, Eric Troncy

**Affiliations:** 1 Groupe de Recherche en Pharmacologie Animale du Québec (GREPAQ), Université de Montréal, Saint-Hyacinthe, QC, Canada; 2 Department of Clinical Sciences, Faculty of Veterinary Medicine, Université de Montréal, Saint-Hyacinthe, QC, Canada; 3 Osteoarthritis Research Unit, University of Montreal Hospital Research Center (CRCHUM), Montreal, QC, Canada

**Keywords:** electrodiagnostic tests, endogenous pain control, facilitation, inhibition, osteoarthritis, sedation

## Abstract

Although chronic pain in feline osteoarthritis involves neuro-sensitization, the associated sensory changes have not been studied. Cats with osteoarthritis (*n* = 20) and healthy controls (*n* = 6) were assessed for peripheral (paw withdrawal threshold, PWT) and spinal (response to mechanical temporal summation, RMTS) sensitization, with lower values indicating higher neuro-sensitization. Functional impairment was assessed using the Montreal Instrument for Cat Arthritis Testing, for Use by Veterinarians [MI-CAT(V)], with higher scores indicating greater impairment. Cerebral sensory integration was evaluated using cortical somatosensory evoked potential (CSEP), brainstem auditory evoked response (BAER), and visual evoked potential (VEP) under dexmedetomidine sedation. The amplitude and latency of action potentials were recorded. The Mann–Whitney *U* test and Spearman’s rank correlation (coefficient = *ρ*) were applied (*α* = 0.05). Cats with pain showed increased CSEP and BAER latency and amplitude (*p* < 0.032) but decreased VEP latency and increased amplitude (*p* < 0.038) compared to healthy cats. The PWT was negatively correlated with CSEP latency and amplitude (*ρ* = −0.51 to −0.43, *p* < 0.037) and BAER latency (*ρ* = −0.80 to −0.60, *p* < 0.039), while the RMTS was negatively correlated with CSEP latency (*ρ* = −0.48, *p* = 0.017). In contrast, the PWT (*ρ* = 0.71 to 0.76, *p* < 0.010) was positively correlated with VEP latency. The MI-CAT(V) score correlated with CSEP amplitude (*ρ* = 0.62, *p* = 0.006), BAER latency (*ρ* = 0.72 to 0.90, *p* < 0.008), and VEP latency (*ρ* = −0.73 to −0.65, *p* = 0.023). Sensory processing is altered in cats with osteoarthritis-associated chronic pain, suggesting differential inhibitory control mechanisms.

## 1 Introduction

Feline chronic pain can lead to the development of peripheral and central sensitization, characterized by allodynia and hyperalgesia (1). This phenomenon of neuroplasticity associated with an increased pain experience, even with innocuous tactile stimulation (2, 3), can be measured using quantitative sensory testing (QST) (4). Neuro-sensitization can affect sensory modalities beyond touch, as demonstrated by correlations between sensitization in osteoarthritic (OA) cats and altered tolerance to auditory and olfactory stimuli (5). Indeed, in a recent behavioral study, the most sensitized cats displayed higher auditory tolerance to repeated repulsive ultrasound and lower tolerance to unpleasant grapefruit odor exposure (5). These findings suggest that chronic pain in OA cats can be associated with broader alterations in sensory perception. However, these results are based on behavioral assessments, which carry a potential subjectivity bias, and were obtained without comparison to healthy cats.

Impairment in sensory perception can be the consequence of transmission and/or integration dysfunction, which can be assessed using objective electrodiagnostic tests. Sensory transmission can be evaluated through nerve conduction studies (6, 7). Alterations in nerve conduction have been reported in OA-associated pain and neuro-sensitization, including decreased conduction velocity and amplitude, suggesting impaired peripheral nerve integrity (8–10). Sensory stimulus integration can be tested using electroencephalography (EEG) and the analysis of evoked potentials (11). An evoked potential (EP) represents the averaged electrical responses of neurons following a sensory stimulus delivered at a specific intensity and frequency (11). In humans with chronic pain, somatosensory evoked potentials (SEPs) recorded at the cortical level have shown increased latency and decreased amplitude (12), whereas findings for auditory evoked potentials (AEPs) are inconsistent, including reports of decreased latency (13, 14). Visual evoked potentials (VEPs), when assessed during cognitive tasks, have been associated with increased amplitude (15). Few older studies in companion animals have established reference values for cortical SEPs (CSEPs) (16), brainstem auditory evoked responses (BAERs) (17), and VEPs (17, 18). More recently, EPs have been used to investigate cross-modal plasticity mechanisms during deafness, showing activation of auditory cortical areas by visual and somatosensory stimuli (19, 20). To date, the effects of feline chronic pain on standardized EPs have not been investigated. Characterizing alterations in sensory integration is particularly important for understanding the extent of neuroplasticity in feline OA, for facilitating positive environmental adaptations, and ultimately for developing innovative and objective methods to assess neuro-sensitization, which remains challenging in clinical settings.

The aim of this study was to explore sensory changes associated with feline OA using electrodiagnostic methods. We hypothesized that (*i*) cats affected by OA chronic pain would demonstrate changes in CSEP, BAER, and VEP latencies and amplitudes compared to healthy controls, and (*ii*) these changes would correlate with neuro-sensitization assessed by QST and with functional impairment, serving as markers of OA severity.

## 2 Materials and methods

### 2.1 Animal selection and housing

The study was approved by the Institutional Animal Care and Use Committee of ArthroLab Inc. (#A196-ART22F) and the Université de Montréal Faculty of Veterinary Medicine (#CEUA-Rech-2114). Healthy neutered cats (*n* = 6, including two female cats) and neutered cats affected by OA chronic pain (*n* = 20, including seven female cats) from the ArthroLab Inc. colony (sourced from Canadian shelters and radiographically confirmed for OA) were included in the study. Their care and handling adhered to the guidelines of the Canadian Council on Animal Care, Guide to the Care and Use of Experimental Animals, Vol. 1, 2nd Edition (1993, revised in Feb. 2017). All procedures adhered to the ARRIVE guidelines for reporting animal research. All healthy cats (*n* = 6) were evaluated for each outcome; they had a mean age of 2 years [1–4] and a mean body weight of 4.46 kg [3.70–5.15]. The characteristics of cats affected by OA are presented in the following sections corresponding to each assessment. Radiographic screening for OA was performed by a board-certified veterinary surgeon (BL) (21, 22). All cats were otherwise healthy (except for OA) based on physical and neurological examinations performed by a board-certified veterinary neurologist (AC) and the absence of abnormal findings on complete blood count and serum chemistry panels. Cats were group-housed in rooms with controlled lighting, temperature, and humidity and provided with positive environmental enrichment. They were fed a veterinary-quality diet twice daily according to the manufacturer’s recommendations, and fresh water was provided *ad libitum*.

### 2.2 Pain outcomes

#### 2.2.1 Quantitative sensory testing

The somatosensory profile was characterized using two QST methods. The paw withdrawal threshold (PWT) was used to assess peripheral sensitization, while the response to mechanical temporal summation (RMTS) was used to evaluate spinal sensitization (1). Both tests were performed as previously described (see the four appendices of (4) for method illustrations). Lower PWT values or fewer tolerated RMTS stimulations were indicative of an increase in peripheral and spinal sensitization, respectively.

#### 2.2.2 Functional assessment

Functional impairment was assessed using the validated Montreal Instrument for Cat Arthritis Testing, for Use by Veterinarians [MI-CAT(V)] (22–26). It includes four sub-sections: Body posture, gait analysis, obstacle passage, and global distance examination. MI-CAT(V) scores (expressed as a percentage of functional alterations) were determined blindly for healthy and OA cats. Higher scores indicated greater functional impairment.

### 2.3 Cortical somatosensory evoked potential (CSEP)

Healthy and OA cats [*n* = 20; mean age 11 years (6–16) and mean body weight 4.45 kg (3.32–5.77)] were evaluated under sedation using dexmedetomidine (Dexdomitor^®^, 0.5 mg/mL, 0.02 mg/kg; Zoetis Canada, Kirkland, QC, Canada) administered subcutaneously, and sedation was reversed by atipamezole, which was injected intramuscularly (Antisedan, 5.0 mg/mL, 0.10 mg/kg; Zoetis Canada, Kirkland, QC, Canada) at the end of the procedure. Routinely used in clinical electrodiagnosis, dexmedetomidine is recognized for producing no artifacts in EP recordings, eliminating movement interference, and being reversible. Somatosensory EPs were recorded using the Sierra II Wedge/EP^®^ system (Caldwell Industries, Kennewick, WA, USA). Cats were placed on their lateral side on a heating pad (Veterinary Heat Pump Patch^®^ / HTP-1500^®^, Adroit Medical Systems, Loudon, TN, USA) to ensure that the body temperature remains above 37.5 °C. Electrical impulses were delivered using a stimulation electrode placed parallel to the left tibia on the medial side (shaved), proximal to the medial malleolus. A total of three subdermal needle electrodes were placed as follows: The active electrode at Cz, the reference electrode at Fz, and the ground electrode between the stimulating electrode and the popliteal region. Stimulations were delivered at 0.5 Hz with an amplitude of 4 mA for a total of 300 stimulations, performed in duplicate (15 min apart) (16, 27). Impedance was maintained below 5 kΩ, and the peaks were identified as previously described (see Appendix 1A for a tracing example and Appendix 1C for electrode placement) (28).

### 2.4 Flash – visual evoked potential (VEP)

Healthy and OA cats [*n* = 6, mean age 12 years (11–14) and mean body weight 4.45 kg (3.36–5.38)] were evaluated for VEPs using the RETIport^®^ system (Roland Consult, Brandenburg an der Havel, Germany) under sedation with dexmedetomidine. A light flash was delivered at 3 cd.s/m^2^ and 1.3 Hz for a total of 300 stimulations (17). The right eye was assessed first, followed by the left eye, with both eyes tested in duplicate (10 min apart). No pupil dilatator was used. The visual response was recorded using three subdermal needle electrodes: The active electrode at Cz, the reference electrode outside the ear canal (ipsilateral side of the stimulated eye), and the ground electrode between the scapulae (29, 30). Impedance was maintained below 5 kΩ, and peak identification was performed as previously described (see Appendix 1B for a tracing example and Appendix 1D for electrode placement) (17).

### 2.5 Brainstem auditory evoked response (BAER)

Brainstem auditory evoked responses were recorded on the same day, after VEP assessment, in sedated healthy and OA cats [*n* = 6, mean age 12 years (11–14) and mean body weight 4.45 kg (3.36–5.38)] using the Sierra II Wedge/EP^®^ system (Caldwell Industries, Kennewick, WA, USA). A click sound was delivered at 11.33 Hz and 90 dB for a total of 500 stimulations, as previously described in cats (17). The BAER was recorded using three subdermal needle electrodes: The active electrode at Cz, the reference electrode outside the ear canal (mastoid reference at the base of the stimulated ear), and the ground electrode between the scapulae. Impedance was maintained below 5 kΩ, and peak identification was performed as previously described (see Appendix 2A for a tracing example and Appendix 2B for electrode placement) (17).

### 2.6 Statistical analysis

The amplitude and latency of each evoked potential were recorded, with the first upward peak designated as P1 and the first downward peak as N1. Repeatability for CSEPs and VEPs, both performed in duplicate, was evaluated using intraclass correlation coefficient (ICC) estimates and their 95% confident intervals (95% CIs) based on a mean-rating, absolute agreement, two-way mixed-effects model (31). Comparisons between healthy and OA cats were performed using the Mann–Whitney *U* test. Correlations between EPs and pain outcomes were calculated using Spearman’s rank correlation. ICC values were interpreted as follows: < 0.50, poor; 0.50–0.75, moderate; 0.75–0.90, good; and > 0.90, excellent reliability. Spearman correlation coefficients (Rho, *ρ*) were interpreted as follows: < 0.40, weak; 0.40–0.69, moderate; 0.70–0.89, strong; and > 0.90, excellent correlation (31, 32). The chosen sample size (*n* = 6–20) was appropriate for a preliminary comparative proof-of-concept study on cerebral sensory changes associated with feline OA (33). The significant threshold was set at *α* = 0.05. Data were analyzed using two software packages: IBM^®^ SPSS^®^ Statistics (IBM Corp. Version 29.0.2.0, Armonk, NY, USA) and JMP^®^ Pro (JMP Statistical Discovery LLC. Version 18.0.2., Cary, NC, USA).

## 3 Results

### 3.1 Cortical somatosensory evoked potential

#### 3.1.1 Repeatability

Considering all cats (healthy, *n* = 6); and with OA chronic pain, *n* = 18—noting a technical issue for (*n* = 2), the two evaluations were repeatable for all latencies (ICC > 0.67 [0.22–0.86], *p* < 0.006) and for P2–N2 and P3–N3 amplitudes (ICC > 0.81 [0.53–0.91], *p* < 0.001) (Appendix 3). Therefore, the mean value was used for the subsequent analysis.

#### 3.1.2 Specificity

Cats affected by OA chronic pain showed an increase in P2 latency (+3%, *p* = 0.047) compared to healthy cats (Table 1).

**Table 1:** Descriptive statistics of cortical somatosensory evoked potentials.

Outcomes	Healthy	*n*	OA	*n*	OA *vs*. Healthy	
	Median [min–max]		Median [min–max]		% change	*p* value
P1 latency (ms)	10.15 [4.35–10.40]	5	8.80 [4.15–11.70]	16	−13%	0.905
N1 latency (ms)	12.17 [8.00–12.67]	5	11.82 [9.01–14.31]	16	−3%	0.398
P1–N1 amplitude (μV)	0.13 [0.04–0.86]	5	0.22 [0.10–2.35]	16	+69%	0.548
P2 latency (ms)	14.18 [12.05–14.50]	6	14.58 [11.25–17.95]	18	+3%	***0.047***
N2 latency (ms)	17.23 [16.33–17.80]	6	17.79 [13.63–20.84]	18	+3%	0.343
P2–N2 amplitude (μV)	1.17 [0.37–1.64]	6	0.50 [0.09–2.94]	18	−57%	0.156
P3 latency (ms)	22.43 [19.55–24.70]	6	22.40 [20.20–24.75]	18	−0%	1.000
N3 latency (ms)	27.23 [24.67–31.25]	6	28.89 [25.68–36.72]	18	+6%	0.280
P3–N3 amplitude (μV)	1.05 [0.64–1.15]	6	2.25 [0.32–7.93]	18	+115%	0.119

The latency and amplitude of each peak are shown for both healthy and OA cats. The percentage change between both conditions and its associated statistical significance are reported in the last column. OA, osteoarthritic; *n* = number of cats with the reported peak.

#### 3.1.3 Correlation with pain outcomes

P2 latency demonstrated a moderate negative correlation with the RMTS (*ρ* = −0.48, *p* = 0.017). A similar association was observed between N3 latency and the forelimb PWT (*ρ* = −0.43, *p* = 0.037). P3–N3 amplitude was moderately correlated with the MI-CAT(V) (*ρ* = 0.62, *p* = 0.006) and forelimb PWT (*ρ* = −0.51, *p* = 0.012). In other words, an increase in P2 and N3 latencies was associated with greater neuro-sensitization, while an increase in P3–N3 amplitude was associated with higher functional impairment and neuro-sensitization.

### 3.2 Flash – visual evoked potential

#### 3.2.1 Repeatability

All peaks were observed in OA cats (*n* = 6), while only P1 and N2 were consistently recorded in all healthy cats (*n* = 6). Considering all OA and healthy cats, the right and left P1 and N1 latencies, left P2 and N2 latencies, right and left P2–N2 amplitudes, and left P3–N3 amplitude demonstrated good repeatability (ICC > 0.80 [0.32–0.94], *p* < 0.011) (Appendix 4). The mean of the duplicate was used, and the averaged right and left VEPs were reliable for P1, N1, P2, N2, P3, and N3 latencies (ICC > 0.71 [−0.03–0.92], *p* < 0.029) and P2–N2 amplitude (ICC = 0.79 [0.13–0.95], *p* = 0.020).

#### 3.2.2 Specificity

The mean of the right- and left-eye assessments was used for the subsequent analysis. P1, N1, P2, N2, and P3 latencies were, on average, 22% shorter in OA cats compared to healthy cats (*p* < 0.038). Conversely, N1–P2 amplitude was higher in OA cats than in healthy cats (*p* = 0.019) (Table 2).

**Table 2:** Descriptive statistics of visual evoked potentials.

Outcomes	Healthy	*n*	OA	*n*	OA *vs*. Healthy	
	Median [min–max]		Median [min–max]		% change	*p* value
P1 latency (ms)	40.50 [32.00–43.50]	6	31.25 [29.00–36.50]	6	−23%	***0.009***
N1 latency (ms)	57.63 [55.00–91.00]	4	44.25 [40.15–56.75]	6	−23%	***0.019***
P1–N1 amplitude (μV)	9.00 [7.62–19.14]	4	3.15 [0.64–19.53]	6	−65%	0.114
P2 latency (ms)	68.63 [64.00–102.00]	4	61.25 [56.00–71.75]	6	−11%	***0.038***
N1–P2 amplitude (μV)	1.85 [1.20–2.91]	4	3.97 [2.67–4.60]	6	+115%	***0.019***
N2 latency (ms)	117.25 [114.25–133.50]	6	90.50 [79.80–99.75]	6	−23%	***0.002***
P2–N2 amplitude (μV)	14.38 [4.14–50.55]	4	4.69 [3.04–7.53]	6	−67%	0.257
P3 latency (ms)	164.88 [163.00–166.75]	2	117.25 [108.45–136.00]	6	−29%	0.071
N3 latency (ms)	205.00 [185.50–224.50]	2	157.88 [137.50–188.00]	6	−23%	0.143
P3–N3 amplitude (μV)	3.39 [1.75–5.04]	2	4.25 [0.96–7.31]	6	+25%	1.000

The latency and amplitude of each peak are shown for both healthy and OA cats. The percentage change between both conditions and its associated statistical significance are reported in the last column. OA, osteoarthritic; *n* = number of cats with the reported peak.

#### 3.2.3 Correlation with pain outcomes

Values of P1 and N2 latencies were retained for subsequent correlation analyses, as all healthy and OA cats showed these peaks. P1 latency demonstrated a strong negative correlation with the MI-CAT(V) (*ρ* = −0.73, *p* = 0.008) and a positive correlation with the hindlimb PWT (*ρ* = 0.76, *p* = 0.004). N2 latency was similarly correlated with the MI-CAT(V) (*ρ* = −0.65, *p* = 0.023) and the forelimb PWT (*ρ* = 0.71, *p* = 0.010). Lower P1 and N2 latencies were associated with greater functional impairment and neuro-sensitization.

### 3.3 Brainstem auditory evoked response

#### 3.3.1 Specificity

Right and left BAERs were similar for each latency and I–Ia amplitudes (ICC > 0.58 [0.03–0.87], *p* < 0.033); therefore, the mean of both sides was used for subsequent analysis (Table 3). Osteoarthritic cats exhibited, on average, 20% longer latency for each peak (*p* < 0.020) compared to healthy cats. The amplitude did not differ based on pain status, although OA cats tended to show lower V–Va amplitudes (−25%, *p* = 0.093) (see Appendix 2A for an example of the tracing).

**Table 3:** Descriptive statistics of brainstem auditory evoked potentials.

Outcomes	Healthy	*n*	OA	*n*	OA *vs*. Healthy	
	Median [min–max]		Median [min–max]		% change	*p* value
I latency (ms)	0.89 [0.72–1.03]	6	1.19 [0.89–1.49]	6	+34%	***0.020***
II latency (ms)	1.58 [1.52–1.64]	6	2.00 [1.66–2.36]	6	+26%	***0.005***
III latency (ms)	2.32 [2.23–2.59]	6	2.66 [2.65–3.20]	6	+15%	***0.005***
IV latency (ms)	3.09 [2.94–3.54]	6	3.49 [3.47–4.30]	6	+13%	***0.013***
V latency (ms)	3.84 [3.53–4.56]	6	4.86 [4.49–5.58]	6	+27%	***0.013***
I–Ia amplitude (μV)	4.37 [0.82–7.01]	6	4.41 [2.90–9.91]	6	+1%	0.936
V–Va amplitude (μV)	1.83 [0.53–2.78]	6	1.37 [0.24–1.67]	6	−25%	0.093

The latency and amplitude of each peak are shown for both healthy and OA cats. The percentage change between both conditions and its associated statistical significance are reported in the last column. OA, osteoarthritic; *n* = number of cats with the reported peak.

#### 3.3.2 Correlation with pain outcomes

BAER latencies from I to IV were negatively correlated with the forelimb PWT (*ρ* = −0.64 to −0.60, *p* < 0.039) (Appendix 2C) and hindlimb PWT (*ρ* = −0.80 to −0.63, *p* < 0.028), while peak latencies from I to V were positively correlated with the MI-CAT(V) (*ρ* = 0.72 to 0.90, *p* < 0.008) (Appendix 2C). Longer BAER latencies were associated with greater functional impairment and neuro-sensitization.

## 4 Discussion

This study provides the first evidence of sensory changes associated with feline OA chronic pain using sensory evoked potentials. Cats affected by OA demonstrated increased CSEP and BAER latencies, decreased VEP latency, and increased amplitudes across all three tested sensory modalities compared to healthy cats. Moreover, CSEP and VEP latencies demonstrated greater repeatability than amplitude, suggesting that conclusions should be interpreted with caution and primarily based on latency. Increased neuro-sensitization (PWT or RMTS) and functional impairment [MI-CAT(V)] correlated with longer CSEP and BAER latencies (i.e., decreased transmission velocity) but shorter VEP latency (i.e., increased transmission velocity). This study provides reference values for feline CSEPs, VEPs, and BAERs recorded under sedation and highlights alterations in cerebral sensory integration associated with chronic pain, with age as a potential contributing factor.

Consistent with previous studies, left and right VEP and BAER values were comparable (17), and BAER results aligned with previously published values for healthy cats (17). However, VEP latency was prolonged, while CSEP latency was shortened by more than 20 ms compared to previous studies (16, 17). Previous studies have shown that VEPs and CSEPs are more affected by sedation than BAERs (34). Indeed, these changes may be attributed to the use of dexmedetomidine in the present study, compared to measurements in conscious animals for VEPs (17) or under anesthesia (xylazine and ketamine) for CSEPs (16). Although the latter has been associated with delayed EPs (35, 36), dexmedetomidine has been reported to have minimal effects on such measures (37). Alternatively, differences in VEP latency may result from variations in electrode placement. In earlier studies, the active electrode was placed at the level of the nuchal crest, whereas Cz was used in this study to record over the somatosensory cortex (17, 38). Further investigations using dexmedetomidine are needed to enable more accurate comparisons.

In chronic pain conditions, signal transmission differed across sensory modalities compared to healthy cats. Cats affected by OA showed delayed CSEP P2 latency, which was correlated with greater spinal sensitization. P3–N3 amplitudes were positively correlated with both peripheral sensitization and functional impairment. CSEP peaks result from stimulation of large myelinated sensory afferents (Aβ), which are implicated in allodynia (28). With our recording configuration, P1 is suspected to originate from the lateral and medial thalamic nuclei or thalamocortical projections, N1 and P2 from the sensorimotor area, and N2 and P3 from association areas (28). The delay in latency could be attributed to demyelination, leading to slower transmission to the sensorimotor area, as supported by recently demonstrated impaired nerve conduction, suggesting demyelination in old OA cats (8, 10) and evidence of bone–brain crosstalk and neuroinflammation in human OA (39). The change in amplitude has previously been correlated with spontaneous activity of spinal neurons, supporting facilitation pathways occurring during chronic pain (40, 41). These findings suggest a compensatory mechanism, with altered transmission velocity being counterbalanced by enhanced effectiveness of the action potential. As observed for CSEPs, all BAER peak latencies were delayed in OA cats compared to healthy cats and were further prolonged in individuals with greater peripheral sensitization (PWT) and functional impairment (MI-CAT(V)). Physiologically, peak I is generated by cranial nerve VIII entering the brain, while the later peaks are generated in the brainstem (42, 43). Our results are consistent with a previous study demonstrating increased tolerance to auditory stimuli in cats with neuro-sensitization (5) but contrast with previous findings in humans with chronic pain experiencing hyperacusis (44), which is characterized by shorter BAER latency (45). Hyperacusis corresponds to enhanced auditory perception linked to reduced inhibition originating in the brainstem (44), suggesting that OA cats in this study, by contrast, may have retained a functional inhibitory system.

For VEPs, all peaks were observed in OA cats, while only P1 and N2 were consistently recorded in healthy cats, in agreement with previous findings in dogs (46). The origin of the VEP has been described in dogs, with early peaks (P1 and N1) representing potentials from the retina to the optic tract and P2 and N2 arising from the visual cortex (47). The P3 component is thought to be influenced by cognitive processing and has been more extensively described in humans (15). Overall, VEP recordings revealed a more complex waveform in OA cats, with additional peaks and shortened P1 and N2 latencies compared to healthy cats. These findings may suggest facilitated visual processing, potentially related to increased vigilance, enhanced central afferent excitability, or modality-specific modulation of inhibitory pathways in chronic pain (48). In contrast to the BAER results, which indicated delayed processing, the VEP changes support the concept of differential sensory integration across modalities rather than a uniform alteration of central inhibition in cats with chronic pain.

These differences in cortical sensory integration can be explained by neuro-sensitization, reflected in a dynamic modulatory balance between inhibitory and facilitatory endogenous pain controls (49). In this study, neuro-sensitization had an inhibitory effect on CSEPs and BAERs but a facilitatory influence on VEPs. Visual, auditory, and somatosensory pathways converge at the level of the brainstem (rostral colliculus for visual reflexes and caudal colliculus for audition) (50) and the thalamus (lateral geniculate nucleus for visual perception and medial geniculate nucleus for audition) (51), both of which are implicated in pain modulation (52, 53). Indeed, inhibitory pathways involve brainstem structures such as the *locus coeruleus* (LC), rostral ventromedial medulla (RVM), and periaqueductal gray (PAG) matter (52). Although auditory and somatosensory pathways involve brainstem relays, the main visual pathway does not, which may explain the observed latency delays, potentially resulting from brainstem-mediated inhibition (54). This is further supported by the presence of inhibitory neurotransmitters such as gamma-aminobutyric acid (GABA) in the cochlea (55). These findings support the hypothesis that inhibitory pathways are functional and active in OA cats, leading to decreased somatosensory and auditory input, while visual stimulation processing is facilitated rather than inhibited. Nevertheless, the pupillary reflex involves the pretectal nucleus and the oculomotor parasympathetic nucleus in the brainstem and can be modulated by pain facilitation or inhibition, particularly as part of autonomic nervous system function (56). Pupillary dilatation is sympathetically mediated in awake patients and can be used to evaluate sympathetic stimulation associated with pain. This rationale has led to the development of an infrared pupillometer, an easy-to-use, non-invasive technique to quantify nociception and monitor opioid effects to reduce their adverse effects in human perioperative medicine (57). A recent translational healthy human and rat study did not identify changes in pupillometric measures during temporal summation (i.e., facilitation) or during the conditioned pain modulation (CPM) paradigm (i.e., inhibition) compared to a unique nociceptive stimulus (58). However, the study did not compare measures to a non-noxious baseline, nor did it examine correlations between the intensity of facilitation/inhibition and pupillometric measures. Indeed, pupillometry has been shown to objectively reflect acute pain responses, such as pain relief through CPM testing in healthy humans (59). Notably, the CPM effect calculated from subjective pain ratings and objective pupillometry measurements was not correlated, suggesting that the two methods assess different aspects of pain perception (59). These results are consistent with the present findings, in which VEPs were not associated with inhibition (CPM). In veterinary medicine, although limited studies have been performed, pupillometric reference values have been established in 126 healthy dogs (60). In dogs undergoing ovariohysterectomy, infrared pupillometry has been suggested as a valuable method for evaluating nociception. In a concurrent validation situation, both pupillometry and the short-form of the Glasgow Composite Pain Scale—a validated clinical metrology instrument for acute pain—reported similar analgesic effects when cannabidiol was administered pre-emptively, either alone and in combination with meloxicam (61). Pupillometric analysis could be useful to determine concordance with CSEPs and BAERs. Facilitating visual information may represent a compensatory mechanism aimed at improving the visual perception of a movement that may cause pain. This phenomenon has been demonstrated in human patients with neck pain. Indeed, individuals reported increased pain when they were visually led to believe that they were turning their heads, suggesting that vision influences pain perception (62). Another hypothesis involves a phylogenetic distinction that may account for differences in neural damage. The optic nerve (transmitting VEPs) is an extension of the central nervous system and is myelinated by oligodendrocytes, while the vestibulocochlear cranial nerve (transmitting peak I BAERs) and the nerves innervating the limbs (CSEPs) belong to the peripheral nervous system and are myelinated by Schwann cells. These structural and cellular differences suggest that peripheral nerves and the central nervous system may be susceptible to distinct neurodegenerative mechanisms, either during neuronal aging or chronic pain. These mechanisms have been implicated in axon demyelination in older cats with OA (8, 10) and in a porcine model of neuropathic pain (63). This study provides a foundation for further investigations. CSEPs and BAERs could potentially serve as markers of inhibitory pathway function or neurodegenerative impairment, while VEPs could be a marker of facilitation. Further studies are needed to explore the balance between facilitation and inhibition in chronic pain conditions and their impact on each sensory modality.

A major limitation of this study is the age of the cats, as cats affected by OA were older than healthy controls. Very few studies have previously examined the effects of aging on EPs. In cats older than 12 years, BAER latency was increased (64), consistent with our results. However, aging in dogs was associated with decreased VEP amplitude and increased latency (65), which contrasts with our results. No information on animal health status has been provided in these studies, and no studies have evaluated age-related changes in VEPs in cats. Given the longer lifespan of cats compared to dogs, it is possible that age-related VEP alterations occur later in cats and that species-specific aging patterns may contribute to these differences. In humans, aging was associated with increased amplitude and prolonged CSEP, VEP, and BAER latencies, as well as enhanced neural activity from one sensory domain to the other. It cannot be ruled out that some older adults in this study were also affected by chronic pain (66). These findings in dogs and humans were only partially reflected in the present feline study, as VEPs did not mirror the changes observed in CSEPs and BAERs. The alterations observed in cats were correlated with QST and functional impairment, suggesting that our results are more likely attributable to chronic pain than aging alone. Further studies are needed to clarify the effects of age on EPs and chronic pain in cats. In addition, a comparative study between conventional VEPs and steady-state VEPs may be valuable to determine whether the latter provides different or additional insights compared to traditional VEP recordings in OA and healthy cats (67).

In conclusion, cortical sensory integration changes occurring during chronic pain in cats were recorded for the first time using standardized EPs. Although CSEPs and BAERs exhibited slower signal transmission, VEPs demonstrated faster signal transmission as functional impairment and neuro-sensitization increased. These findings suggest modality-specific differences in inhibitory control or differential vulnerability to neuro-sensitization between auditory/somatosensory and optic pathways. Although further studies are needed, there is potential to assess inhibitory pathway function in a veterinary context. Indeed, QST is not yet applicable in clinical settings, unlike EPs. The use of the latter makes it possible to assess the imbalance between endogenous pain control and, ultimately, to guide personalized therapy.

## Data Availability

The raw data supporting the conclusions of this article will be made available by the authors, without undue reservation.
