# Supplementary material for: Somatosensory and visual evoked potentials and brainstem auditory evoked responses in osteoarthritic cats with chronic pain – a comparative study
Source: Front Vet Sci. 2026 Apr 10;13:1794107. doi: 10.3389/fvets.2026.1794107 (PMC13106083; doi:10.3389/fvets.2026.1794107)
Supplement: Supplementary file 1 [file Supplementary_File_1.pdf]

Appendix 1 – Example of tracing and electrode placement for SEP (A. and C.) and VEP (B. and D.). Head diagram and cortical electrode placement based on Lewis *et al*., 2011. Ref, reference.

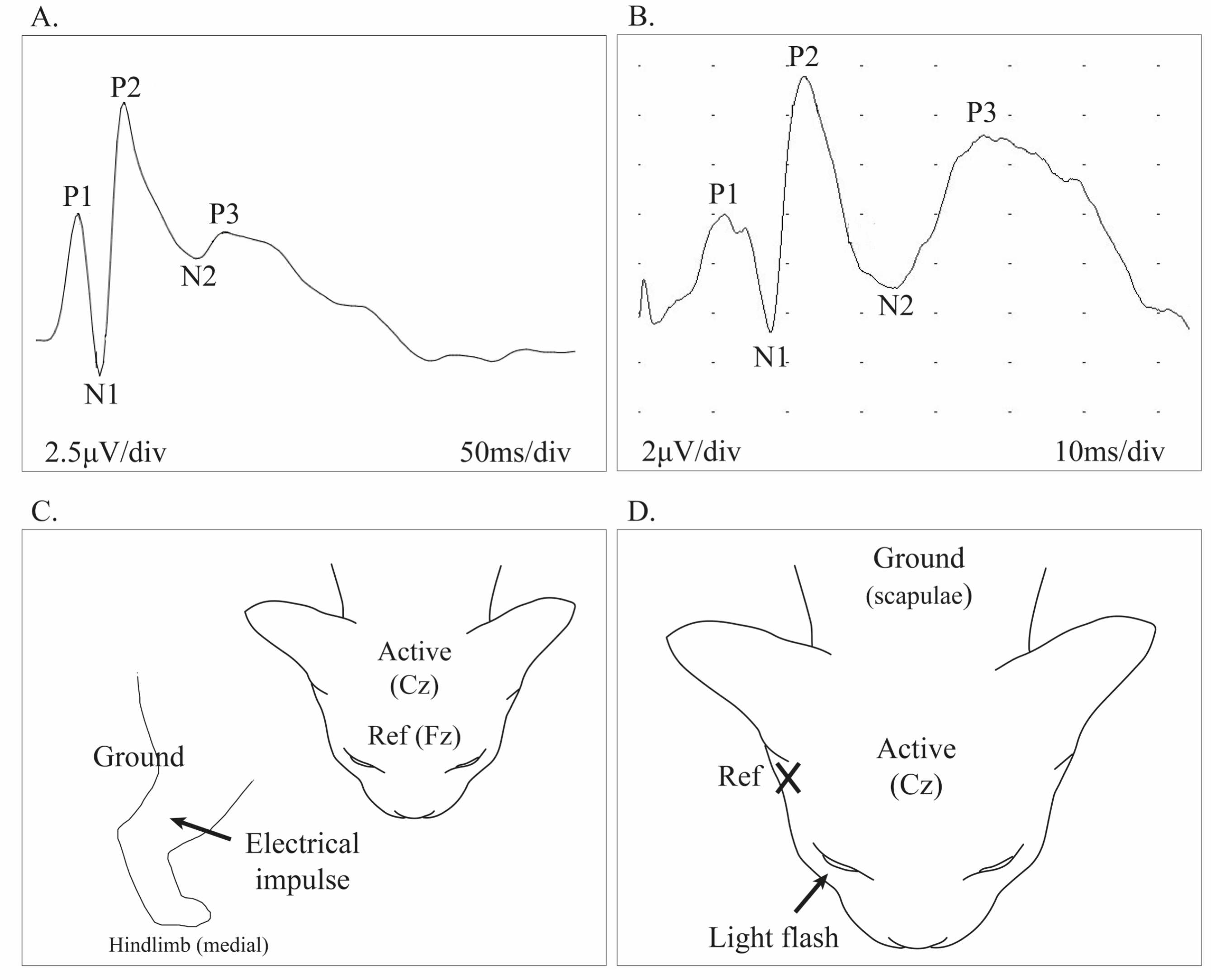

Lewis MJ, Williams DC, Vite CH. Evaluation of the electroencephalogram in young cats. *Am J Vet Res*. (2011) 72(3):391–7. doi: 10.2460/ajvr.72.3.391
