# Supplementary material for: Somatosensory and visual evoked potentials and brainstem auditory evoked responses in osteoarthritic cats with chronic pain – a comparative study
Source: Front Vet Sci. 2026 Apr 10;13:1794107. doi: 10.3389/fvets.2026.1794107 (PMC13106083; doi:10.3389/fvets.2026.1794107)
Supplement: Supplementary file 3 [file Supplementary_File_3.pdf]

Appendix 3 – Intraclass coefficients correlations and their 95% confidence intervals for somatosensory evoked potentials of all cats [healthy (*n* = 6) and OA chronic pain (*n* = 18)].

	**ICC [95% CI]**	***P*-value**	***n***
P1 latency (ms)	0.85 [0.58–0.94]	***< 0.001***	18
N1 latency (ms)	0.77 [0.38–0.91]	***0.002***	18
P1-N1 amplitude (μV)	0.20 [−1.22–0.71]	0.328	18
P2 latency (ms)	0.93 [0.84–0.97]	***< 0.001***	23
N2 latency (ms)	0.83 [0.60–0.93]	***< 0.001***	23
P2-N2 amplitude (μV)	0.81 [0.53–0.91]	***< 0.001***	23
P3 latency (ms)	0.70 [0.31–0.87]	***0.003***	23
N3 latency (ms)	0.67 [0.22–0.86]	***0.006***	23
P3-N3 amplitude (μV)	0.94 [0.85–0.97]	***< 0.001***	23
