# Supplementary material for: Somatosensory and visual evoked potentials and brainstem auditory evoked responses in osteoarthritic cats with chronic pain – a comparative study
Source: Front Vet Sci. 2026 Apr 10;13:1794107. doi: 10.3389/fvets.2026.1794107 (PMC13106083; doi:10.3389/fvets.2026.1794107)
Supplement: Supplementary file 4 [file Supplementary_File_4.pdf]

Appendix 4 – Intraclass coefficients correlations and their 95% confidence intervals for right and left visual evoked potentials of all cats [healthy (*n* = 6) and OA chronic pain (*n* = 6)].

	**Right**			**Left**		
	**ICC [95% CI]**	***P*-value**	***n***	**ICC [95% CI]**	***P*-value**	***n***
P1 latency (ms)	0.91 [0.70–0.97]	**< *0.001***	12	0.94 [0.79–0.98]	**< *0.001***	12
N1 latency (ms)	0.82 [0.29–0.96]	***0.011***	9	0.85 [0.43–0.96]	***0.003***	10
P1-N1 amplitude (μV)	–0.24 [–8.41–0.74]	0.605	9	0.49 [–0.66–0.87]	0.145	10
P2 latency (ms)	0.61 [–0.73–0.91]	0.106	9	0.88 [0.53–0.97]	***0.002***	10
N1-P2 amplitude (μV)	0.50 [–0.75–0.88]	0.153	9	–1.00 [–21.64–0.57]	0.809	10
N2 latency (ms)	0.52 [–0.86–0.87]	0.132	12	0.80 [0.32–0.94]	***0.007***	12
P2-N2 amplitude (μV)	0.93 [0.60–0.99]	**< *0.001***	9	0.98 [0.94–1.00]	**< *0.001***	10
P3 latency (ms)	0.30 [–4.78–0.87]	0.342	8	0.53 [–1.95–0.91]	0.188	8
N3 latency (ms)	0.14 [–7.39–0.84]	0.433	8	0.61 [–0.84–0.92]	0.120	8
P3-N3 amplitude (μV)	0.30 [–2.15–0.86]	0.324	8	0.85 [0.35–0.97]	***0.009***	8
